# Evaluation of Home Blood Pressure Monitoring for Patients with Hypertensive Disorders of Pregnancy: A Rapid Review

**DOI:** 10.3390/healthcare14081102

**Published:** 2026-04-20

**Authors:** Meighan Mary, Sarah Clifford, Andreea A. Creanga

**Affiliations:** 1 International Health Department, Johns Hopkins Bloomberg School of Public Health, Baltimore, MD 21205, USA; 2 School of Medicine, University of Maryland, Baltimore, MD 20201, USA

**Keywords:** pregnancy-induced hypertension, pre-eclampsia, eclampsia, blood pressure monitoring, telehealth

## Abstract

**Background/Objectives**: Hypertensive disorders of pregnancy (HDPs) affect approximately one in seven hospital deliveries in the United States and increase the risk of pregnancy-associated mortality. Home blood pressure monitoring (HBPM) for patients with HDPs has emerged as a model of care poised to improve ascertainment of blood pressure and triage of care during pregnancy and postpartum periods. However, the strength of evidence supporting HBPM approaches has been variable. This rapid review aimed to understand how HBPM approaches for pregnant and postpartum populations with HDPs have been evaluated in order to strengthen future research. **Methods**: Search criteria included peer-reviewed literature in English and French published during 2018–2024 that assessed HBPM approaches for pregnant and postpartum populations in high-income countries. A total of 370 records were screened and reviewed to identify 52 eligible articles. Key study characteristics, methodologies, and outcome measures were extracted. Identified outcome measures were mapped by outcome type (implementation, health service, and client) to assess gaps in evaluation of HBPM approaches. **Results**: A range of study designs were employed to evaluate HBPM approaches: experimental (17%), observational (52%), qualitative (10%), mixed method (10%), and economic (11%) designs. Over a third employed a comparison group, most of which compared HBPM approaches to usual antepartum or postpartum care. Only 11 studies reported on impact outcomes (long-term blood pressure control, adverse maternal and perinatal outcomes). Significant gaps were identified among the implementation outcomes examined. While patient engagement measures were common, assessment of provider adherence and engagement was limited. Hospital admissions and emergency department visits were often employed as proxies to measure HBPM effectiveness, efficiency, and safety. However, no studies adequately reported effectiveness measures for remote patient triage. **Conclusions**: Our results call for improved HBPM metrics to ensure patients are receiving high-quality care responsive to their clinical condition. Future studies on HBPM approaches should prioritize more transparent reporting on health actor engagement. A composite measure including both patient and provider adherence to monitoring and triage processes will provide stronger evidence on the effectiveness of HBPM for pregnant and postpartum patients and share impactful learning for health systems interested in adopting HBPM approaches.

## 1. Introduction

High blood pressure, or hypertension, is a serious health risk for adults across the United States and a key risk factor for heart disease and stroke. Nearly one in two adults in the United States have hypertension, and most (over 92.1 million) do not have their blood pressure (BP) under control [1]. Hypertensive disorders of pregnancy (HDPs) is an umbrella term used to describe diagnoses of gestational hypertension, pre-eclampsia/eclampsia, and chronic hypertension with superimposed pre-eclampsia/eclampsia during pregnancy. HDPs are some of the most common conditions during pregnancy, estimated to affect one in seven hospital deliveries in the United States [2]. Evidence suggests these conditions are on the rise: the prevalence of HDPs among delivery hospitalizations increased by 2.6% during 2017–2019 (13.3% to 15.9%) [3] and the rate of chronic hypertension in pregnancy doubled between 2008 and 2021 (1.8% to 3.7%) [4]. Having an HDP increases the risk of a variety of serious health outcomes, including pregnancy-associated mortality at 1 year postpartum [5]. People with HDPs are also at an increased risk for cardiovascular disease in the future, with recent evidence demonstrating an increased risk of hypertension 10–14 years after delivery for individuals with a high BP in the third trimester [6,7]. However, severe complications and mortality from HDPs are often considered preventable, with clinical guidance emphasizing the role of early identification and timely treatment to prevent poor outcomes [3].

Home blood pressure monitoring (HBPM) (also commonly referred to as remote, self, or telehealth monitoring of blood pressure) has emerged as a model of care poised to improve outcomes for patients with HDPs. In this approach, in addition to usual care, patients take their own blood pressure measurements at home, remotely, or outside of a clinical setting. Models for following-up with these patients vary: in a clinician-led model, the provider remotely receives and assesses data from BP readings to advise the patient, while in a self-monitoring model, the patient receives education on how and when to measure their BP and is responsible for assessing their own measurements with established thresholds at which to seek care.

For non-pregnant patients, out-of-office or HBPM for hypertension management is recommended by many professional organizations, including the American College of Cardiology (ACC) and American Heart Association (AHA) [8]. For pregnant or postpartum patients with an HDP, however, an insufficient evidence base has limited clinical recommendations and implementation guidance for HBPM [9,10]. Systematic reviews underscore the limited evidence assessing the effectiveness of HBPM yet suggest that HBPM is a feasible intervention with the potential to improve ascertainment of BP, patient satisfaction, and promote equity [11,12]. In 2022, noting the low certainty of evidence, the World Health Organization published a conditional recommendation for patients with HDPs suggesting that HBPM should be made available as “an additional option to clinic blood pressure monitoring by health workers during antenatal contacts only” [13]. For individuals at risk for pre-eclampsia, a 2023 American Journal of Obstetrics and Gynecology (AJOG) special report strongly recommended HBPM alongside provision of patient education materials, noting the necessity of a clinical support system to respond to reports of high readings [14].

In 2022, the Maryland Maternal Health Innovation Program (MDMOM) launched a telehealth initiative to address severe hypertension in pregnancy. The initiative seeks to improve BP monitoring for patients at risk and diagnosed with severe hypertension in pregnancy or postpartum through statewide distribution of free Bluetooth BP monitors and cuffs and educational materials [15]. Patients are eligible if they have a high BP reading during their clinic visit (>140/90 mmHG), a diagnosis of an HDP, or chronic hypertension. Once a provider has confirmed the patient meets the eligibility criteria, patients are equipped with a BP monitor and appropriately sized cuff, and are supplied with educational materials detailing how to measure their BP at home and urgent maternal warning signs. Health providers also demonstrate how to use the BP unit and ensure patients complete the telehealth initiative registration information. At a minimum, patients are advised to contact their health provider if they have a home BP reading between 140 and 159/90–109 mmHG. However, to ensure better integration within hospital service delivery workflows across Maryland, the MDMOM team has collaborated with participating birthing hospital leadership to adapt the initiative, giving each institution flexibility to establish their own monitoring and follow-up protocols.

Similarly, as part of the National Institutes of Health’s IMPROVE Initiative, researchers are developing and evaluating HBPM approaches [16]. The IMPROVE Initiative supports research to promote maternal health equity and reduce pregnancy-related morbidity and mortality through the Community Implementation Program (CIP) and the establishment of twelve Maternal Health Research Centers of Excellence across the United States. For example, the Southern Center for Maternal Health Equity, a Maternal Health Research Center of Excellence led by Tulane University, the Praxis Project, and Oschner Health, aims to adapt and assess an enhanced HBPM approach across rural parishes in northern Louisiana.

To address identified evidence gaps and support future design, implementation, and evaluation of these initiatives, we explored how various HBPM approaches could be measured. This paper synthesizes findings from a rapid review employed to understand how researchers have studied and evaluated home blood pressure monitoring approaches for pregnant and postpartum populations with hypertensive disorders.

## 2. Materials and Methods

A rapid review was conducted of peer-reviewed literature that assessed implementation of HBPM interventions for pregnant and postpartum patients [17]. The protocol was registered with the Open Science Foundation (https://osf.io/m8b2e, accessed on 31 December 2024). MEDLINE and Scopus databases were searched electronically in December 2024, employing a search strategy that included keywords related to HBPM and pregnant and postpartum populations (see Supplementary Materials; Table S1). In addition, reference lists of relevant articles were searched for additional literature.

Table 1 outlines the eligibility criteria. The literature was eligible if it assessed HBPM and related approaches for pregnant and postpartum people within high-income countries. Peer-reviewed research, program evaluation, and field report articles published in English or French during 2018–2024 were eligible to assess the most recent methodologies employed to evaluate HBPM approaches. Reviews, letters, commentaries, editorials, protocols, abstracts, posters, and any other article type that did not report original research or evaluation data or was not required to undergo peer review were excluded. Reference lists of relevant reviews were cross-checked to identify additional eligible literature not identified through database searches.

The identified literature was uploaded into Covidence software [18] for screening. MM screened abstracts and conducted subsequent full-text review. SC reviewed approximately 20% of the literature at each stage; no conflicts were identified (100% inter-rater reliability). MM extracted data for all eligible articles using a piloted data extraction template that captured key study characteristics, methodology and outcome measures.

Characteristics of identified HBPM approaches were summarized. In addition, eligible articles were mapped to understand commonly used programmatic inputs, HBPM approaches and activities as well as identify key output, outcome, and impact measures. Outcomes were categorized as implementation, health service delivery, and client measures in alignment with Proctor et al.’s (2011) conceptual model of implementation research [19,20] that incorporates the Institute of Medicine’s six quality improvement aims: efficiency, safety, effectiveness, equity, patient-centeredness, and timeliness (Table 2) [21]. To assess literature and/or knowledge gaps, outcome and impact indicators were mapped by an HBPM approach (i.e., antepartum or postpartum blood pressure monitoring). Risk of bias and quality appraisals were not conducted due to the methodological focus of the review.

## 3. Results

**Figure 1:**
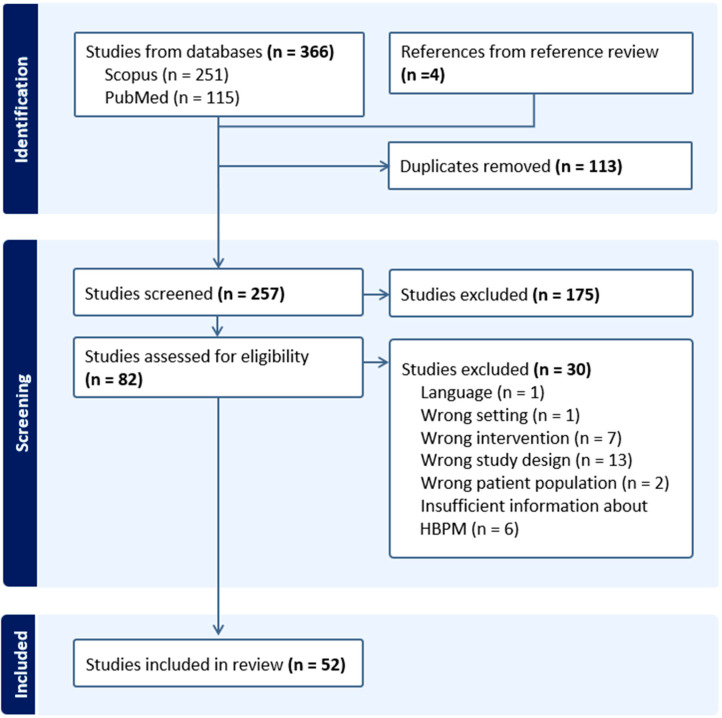
Prisma flow diagram.

A total of 370 peer-reviewed articles were identified, 366 from Scopus and PubMed databases and four from review of reference lists (Figure 1). After removing 113 duplicates, 257 articles underwent screening, and 82 underwent full-text review. At this stage, 30 articles were excluded based on study eligibility criteria: one article was in German, one article was reporting on an HBPM approach in a low-income country, seven reported on telehealth interventions that did not include HBPM approaches, 13 were editorials or commentaries that did not report on results from an assessment or study on HBPM, two reported on HBPM approaches with patient populations that were not pregnant or in the postpartum period, and six did not report sufficient information about the HBPM approach to understand how the program was assessed.

A total of 52 peer-reviewed articles were eligible [22,23,24,25,26,27,28,29,30,31,32,33,34,35,36,37,38,39,40,41,42,43,44,45,46,47,48,49,50,51,52,53,54,55,56,57,58,59,60,61,62,63,64,65,66,67,68,69,70,71,72,73], many of which assessed the implementation of the same HBPM approach or program (Table 3). Over half of the studies (*n* = 27; 52%) focused on HBPM approaches within the antepartum period, 40% in the postpartum period (*n* = 21), and 8% in both periods (*n* = 4). Most articles (*n* = 47; 90%) assessed HBPM approaches that employed a digital platform or automated blood pressure monitor to communicate and/or signal patients’ high blood pressure readings to a health provider, electronic medical record, or patient portal. However, some required or had the option for the patient to call, text, or email a health provider (*n* = 5; 10%) to alert of high BP readings. One HBPM approach also provided the option for rapid telehealth appointments as a mechanism to triage high BP readings. In addition, Wilson et al. (2022) and Bisson et al. (2023) reported on national approaches which distributed blood pressure units to interested hospitals and health systems in England and the United States, respectively, and allowed for each recipient to employ their preferred HBPM approach [71,72].

A range of study designs and methodologies were employed to evaluate HBPM approaches (Table 4), including (quasi-)experimental (*n* = 9; 17%), observational (*n* = 27; 52%), qualitative (*n* = 5; 10%), mixed method (*n* = 5; 10%), and economic (*n* = 6; 11%) designs. Over a third (38%) employed a comparison group, most of which compared HBPM approaches to usual care. Primary outcomes varied widely. However, patient fidelity measures (i.e., patient engagement, adherence, persistence with intervention, submission of BP readings, etc.) were most commonly employed in (quasi-)experimental (*n* = 19; 56%) and observational studies (*n* = 13; 37%). Only one study employed provider fidelity outcomes (provider use and interpretation of home BP to manage clinical care) as a primary outcome [70]. Some studies also used health service measures for their primary outcomes: three studies utilized effectiveness measures, including BP control [29,51] and hospital admissions [67], and one study measured the time between randomization and clinic diagnosis of hypertension [28].

Mapping of reported measures demonstrated gaps in key implementation, health service, and client outcomes (Supplementary Materials Tables S2–S4). All articles employing (quasi-)experimental studies of antepartum HPBM approaches reported fidelity, effectiveness, patient-centered, safety, satisfaction, and clinical status outcome measures [25,28,29]. However, similar designs evaluating postpartum approaches did not report patient-centered or clinical status outcomes [49,51,56,57,65,67]. The reporting of outcomes among articles employing observational, qualitative, or mixed method designs varied significantly. Among those evaluating antepartum HBPM approaches, there is a notable shift in study focus from primarily measuring health service and client outcomes to greater inclusion and prioritization of implementation outcomes in more recent years. In comparison, articles evaluating postpartum or combined HBPM approaches have consistently reported on a few select implementations, health services, and client outcomes. Among articles reporting on economic analyses [22,23,30,34,42,69], cost outcomes were systematically reported. In addition, van den Heuvel et al. (2021) reported on health service, client, and impact outcomes [42]. Overall, significant gaps in measuring implementation outcomes across the literature were identified, namely measures of penetration (*n* = 7; 13%), acceptability (*n* = 5; 10%), sustainability (*n* = 3; 6%), and adoption (*n* = 1; 2%). In addition, very few articles reported equity-related health service outcomes (*n* = 7; 13%) and function-related client outcomes (*n* = 2; 4%). Impact outcome measures were more likely to be reported when evaluating antepartum HBPM approaches [22,25,28,29,33,35,40,42,46] compared to postpartum or combined HBPM approaches [51,73]; nonetheless, only 21% of articles (*n* = 11) reported on such measures (e.g., long-term BP control and adverse maternal and perinatal outcomes).

**Figure 2:**
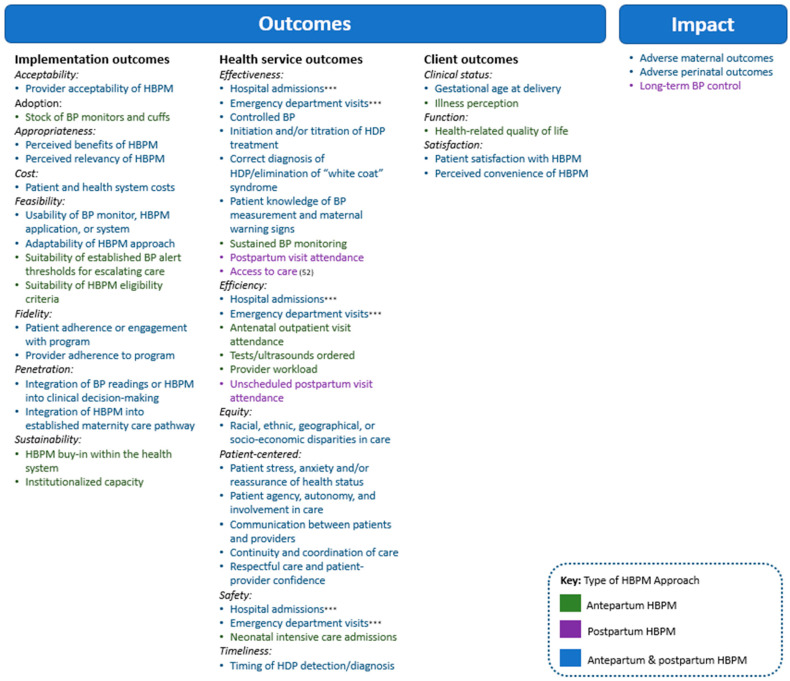
Key HBPM outcome and impact measures identified from peer-reviewed literature. Acronyms: BP: Blood Pressure; EHR: Electronic Health Records; HBPM: Home Blood Pressure Monitoring; HDPs: Hypertensive Disorders of Pregnancy. *** Cross-cutting measures that were employed to measure three domains of health service outcomes: effectiveness, efficiency, and safety.

The breadth of indicators reported within each outcome category was also examined. Figure 2 presents key measures extracted from the literature, distinguishing between outcomes measured to evaluate antepartum and postpartum HBPM approaches. Implementation, health service, and client outcomes were identified along with three primary impact measures: long-term postpartum blood pressure control [51], adverse maternal outcomes [22,25,28,29,33,35,40,42,46,73], and adverse perinatal outcomes [25,28,29,33,35,42].

### 3.1. Implementation Outcomes

Among reported implementation outcomes, fidelity and feasibility measures were most frequently employed using both qualitative and quantitative methodologies to assess antepartum and postpartum HBPM approaches. Fidelity or the adherence to the established HBPM approach was assessed primarily from the patient or user perspective. Most studies (*n* = 34; 65%) measured patient adherence or engagement with the HBPM approach by assessing the number of BP readings conducted by users and whether users sent or reported their BP readings back to a health provider or established platform or portal [25,26,27,28,29,37,38,39,40,45,46,47,48,49,50,52,53,54,55,56,57,58,59,60,61,62,63,65,66,67,70,71,72,73]. However, measurement of provider adherence was less common (*n* =7; 13%) [32,37,43,60,66,70,72]. While most studies reported on how patients engaged with the system, in most studies it was unclear to what extent health providers (1) monitored reporting systems (e.g., checked EHR, provider platforms, or patient portals) to review reported BP readings and (2) effectively triaged patients upon report of high BP.

Measurement of feasibility primarily concerned the usability of the blood pressure unit, HBPM application or portal, and/or overall approach [27,32,38,39,40,41,43,44,45,47,50,52,59,60,61,62,65,66,72]. However, a few studies also assessed the suitability of the eligibility criteria and/or established BP alert thresholds for escalating care (i.e., specified BP reading ranges whereby patients should contact the health system or seek emergency care) for antepartum HBPM approaches [39,43,44]. Lavallee et al. (2024) and Hayden-Robinson et al. (2023) also documented the adaptability of HBPM approaches including how the HBPM approach was adjusted in response to health provider feedback [37,55].

Appropriateness was assessed qualitatively to understand patient and provider views of the relevancy [31,32,38,52,59,72] and benefits [31,41,43,44,45,50,52,59,72] of HBPM approaches and provider acceptability was assessed using mixed methods [27,31,43,50,72]. Only one outcome related to the HBPM adoption was reported: the stock of BP monitors and cuffs for an antepartum HBPM approach [37]. Other possible adoption measures related to the intention or readiness to implement HBPM approaches were not identified within the literature.

Similarly, the breadth of measures employed to assess the penetration and sustainability of HBPM approaches was limited. To assess the extent to which HBPM was integrated within health systems (penetration), six articles (12%) measured the integration of BP readings into clinical decision-making [25,26,31,32,70,72] and two assessed integration within established maternity care pathways [37,72]. Nonetheless, penetration in terms of implementation scale, reach, and/or alignment or linkage to other models of care outside maternal health was not assessed. Measurement of sustainability was only captured via qualitative assessment of health system leadership buy-in [31,43] and institutionalized capacity [37] among articles evaluating antepartum HBPM approaches. Financial sustainability measures and the sustainability of postpartum HBPM approaches were not assessed. However, associated patient and health system costs to implement HBPM systems were documented via cost-minimization and economic analyses [23,24,30,34,42,69].

### 3.2. Health Service Outcomes

The identified HBPM studies largely prioritized assessment of health service outcomes as evidenced by the breadth of measures associated with each of IOM’s six quality improvement outcomes (effectiveness, efficiency, patient-centeredness, safety, timeliness, and equity; Figure 2). A total of nine effectiveness outcomes were identified, including two cross-cutting measures: hospital admissions and emergency department (ED) visits. These two outcomes were employed as proxy measures for (1) effectiveness with a reduction in hospital admissions or ED visits denoting effective triage of patients, (2) (in)efficiency in healthcare consumption, and (3) safety as hospital admissions and/or ED visits serve as an indication of need for higher care. Approximately 40% and 19% of articles reported on hospital admissions and ED visits, respectively.

In addition, across antepartum and postpartum HBPM approaches, BP control (*n* = 15; 29%) [25,26,28,29,40,42,49,53,55,58,61,63,64,66,67] and initiation or titration of HDP treatment (*n* = 13; 25%) [43,49,50,55,56,57,58,59,62,63,65,66,67] were commonly assessed to demonstrate service effectiveness. Sustainment of BP monitoring beyond the program period was also assessed among antepartum HBPM approaches, often including continued home blood pressure measurement in the postpartum period [25,27,33,37]. Effectiveness outcomes specific to postpartum HBPM approaches included postpartum visit attendance [49,52,54,56,62,63,65,73] and qualitative perceptions related to patient access to care [52] as proxies to measure improved engagement with the health system.

Efficiency of HBPM approaches was measured primarily in terms of healthcare consumption. Studies assessing antepartum HBPM measured antenatal outpatient visit attendance [22,33,34,35,37,40,41,42] and the number of tests and ultrasounds ordered [22,33,40,42,43], while postpartum HBPM studies measured unscheduled postpartum visit attendance [65,71]. Perceptions of the change in provider workload were also captured in three antepartum HBPM articles [27,37,43].

Five patient-centered outcomes were reported across antepartum and postpartum HBPM approaches. Over a third of articles (*n* = 18; 35%) captured outcomes related to patient stress, anxiety and/or the reassurance of health status as a result of implementing HBPM [25,27,28,29,32,36,38,39,41,43,44,45,50,61,62,70,71,72]. Outcomes related to patient agency, autonomy, and involvement in care, communication between patients and providers, and continuity and coordination of care were also commonly measured. Only four articles assessed respectful care and patient–provider trust [27,41,50,71].

Excluding the cross-cutting outcome measures, only one outcome was identified for the safety, timeliness, and equity domains. Studies assessing antepartum HBPM approaches reported neonatal intensive care admissions as a safety measure [22,25,28,29,33,35,40,42]. In addition, timeliness was measured by assessing the timing of HDP detection and diagnosis [28,29,40,41,42,43,45,66,67]. However, measurement of timely response and/or triage of reported BP measures was not identified. Lastly, equity measures disaggregating or stratifying service delivery outcomes by race, ethnicity, geography, and/or socioeconomic status were employed to assess disparities in care [46,53,54,57,63,65,72].

### 3.3. Client Outcomes

While many pregnancy and delivery characteristics were reported across the identified articles, few outcomes were identified related to patients’ clinical status and function. For antepartum HBPM approaches, gestational age at delivery was most frequently reported as a proxy for patients’ clinical status [22,25,28,29,33,35,40,42,45,73]. In addition, Tucker et al. (2022) also assessed patients’ illness perceptions while conducting postpartum HBPM [28]. Function outcomes are those that assess patient functioning in daily activities, including in social contexts and for the promotion or prevention of adverse health outcomes. Across the studies, only two articles considered function outcomes with the assessment of patients’ health-related quality of life [28,29].

Patient satisfaction was the most frequently reported client outcome for both antepartum and postpartum HBPM approaches. Almost half of the articles (*n* = 23; 44%) employed quantitative and/or qualitative measures to assess patients’ satisfaction with HBPM. Yet, only three employed validated patient satisfaction scales (25, 44, 45). In addition, the perceived convenience of HBPM was often measured qualitatively in relation to patient satisfaction.

## 4. Discussion

Our rapid review reveals a growing literature base focused on demonstrating the value of HBPM approaches for pregnant and postpartum populations at risk for or diagnosed with HDPs. While earlier studies employed experimental research designs to assess the feasibility, safety, and effectiveness of antepartum and postpartum HBPM, a wide range of methodologies and measures have been utilized over the past five years to strengthen the evidence base, with strong reliance on patient implementation fidelity outcome measures and health service consumption measures as proxies to assess effectiveness (i.e., hospital admissions, ED visits, etc.). Nonetheless, when employing these measures in randomized controlled trials to compare HBPM to usual care, researchers have found little to no difference in primary outcomes between HBPM and usual care groups [25,28,29] or greater patient engagement with HBPM approaches, as expected due to the inherent premise of the intervention [56,57,65].

Schoenthaler et al. (2024) questions whether the lack of positive findings are due to poor implementation or an ineffective intervention [74]. Due to the significant gaps identified in measurement, especially of provider adherence or engagement in HBPM, our findings support this conundrum. In the past five years, only 13% (*n* = 7) of studies documented provider adherence outcomes, none of which employed an experimental or quasi-experimental design nor compared outcomes to usual antepartum or postpartum care. In fact, only four studies quantitatively assessed provider implementation via measures of frequency of provider response or review of elevated home BP readings [60,72], provider-initiated contacts to participating patients [66] and physician use and interpretation of BP measurements to manage clinical care [70].

Beyond the counts of provider engagement with HBPM systems, assessment of effective patient triage upon submission or alert of elevated BP was not captured by any study. Strong assumptions that a documented or “controlled BP” by a predetermined timepoint in the pregnancy or postpartum periods or reductions in hospitalizations and ED visits were employed across the literature as “proof” that HBPM is effective or impactful [28,29,35,40,49,51,56,57,58,65,67]. However, it remains unclear if patients undertaking HBPM are receiving appropriately triaged care that responds to their physiological needs. For example, an increase in hospitalizations or ED visits could simply mean that patients are referred to escalated levels of care to appropriately address their symptoms. Conversely, reduction in healthcare utilization could mean patients’ elevated BP readings are going unnoticed without adequate monitoring and clinical oversight.

As remote monitoring and telehealth interventions become increasingly popular, there is a need to consider how we can look beyond the collected clinical data to understand the pathways through which it is used to inform clinical decisions. Studies on remote triage strategies and HBPM in non-pregnant populations may provide insight on methodological approaches to better capture how implementers can assess risk and direct patients to appropriate levels of care. Vainio et al. (2024) conducted a scoping review to identify performance indicators for telephone triage [75]. Among five identified health service outcomes, three were not assessed in any HBPM literature, namely the accuracy of triage decision (percentage of accurately, over-, and under-triaged patients), severity and urgency of symptoms, and the triage response. In 2019, a systematic review of the effectiveness of remote triage was also conducted by researchers at the Department of Veterans Affairs [76]. Like our results, eligible studies reported on healthcare consumption or service utilization (primary care, ED visits, and hospitalizations), patient satisfaction, and cost; however, some of the literature on remote triage also reported upon case resolution metrics following patient or provider contacts which were not identified in our study.

Assessment of clinical decision-making for appropriate triage of care has also been conducted in HBPM approaches within primary care [77]. For example, Lee et al. (2022) reviewed EHR-documented clinical responses to high BP alerts to assess the frequency and type of clinical action taken [77]. This methodological approach, and others aimed at capturing provider response processes, are crucial to gaining a more nuanced understanding of the mechanisms required for HBPM interventions to reduce adverse maternal and neonatal events and support the experience of care of both pregnant and non-pregnant populations.

Our rapid review is not without limitations. We limited our search to high-income countries to ensure comparability to the United States health system context and functionality. However, in doing so, we may have excluded relevant evidence from low- and middle-income countries with different health systems, HBPM approaches, and related metrics. Exclusion criteria related to the publication language also limited our results to studies from English and Francophone countries. Since we took a rapid review approach, we did not register our protocol.

## 5. Conclusions

Our results call for improved HBPM metrics including the measurement of remote triage to ensure patients are receiving high-quality care responsive to their clinical condition. Future studies on HBPM approaches should prioritize more transparent reporting on health actor engagement. A composite measure including both patient and provider adherence to monitoring and triage processes will provide stronger evidence on HBPM for pregnant and postpartum patients and share impactful learning for other health systems interested in adopting HBPM approaches.

## Figures and Tables

**Table 1:** Literature eligibility criteria.

Concept	Include	Exclude
Population	Pregnant or postpartum people	Individuals who are not pregnant or more than 1 year postpartum
Intervention	Approaches that employ remote, home, or telehealth modalities to monitor patient blood pressure outside the health system	No intervention or approach Ambulatory blood pressure monitoring
Comparison	Any or none	N/A
Outcomes	All	N/A
Geographical scope	High-income countries	Low- and middle-income countries
Time	Published 2018–2024	Published before 2018
Study type	All	N/A
Article type	Research Program evaluation Field reports	Reviews Letters/Commentaries Editorials Protocols Abstracts/Posters Artiles that did not undergo peer review
Language	English or French	All other languages

**Table 2:** Domains of outcome measures.

Concept	Domain	Definition
Implementation Outcomes [19,20]	Acceptability	Perception among implementation stakeholders that HBPM is agreeable, palatable, or satisfactory
	Adoption	The intent to uptake HBPM from the organizational or implementer perspective
	Appropriateness	Perceived fit or relevance of HBPM approach
	Cost	HBPM’s cost-effectiveness, the cost around intervention development, implementation of the intervention, and cost information that can inform adoption decisions
	Feasibility	The extent to which HBPM can be successfully used or implemented
	Fidelity	The degree to which HBPM was implemented as intended, according to guidelines, protocols, or action plans
	Penetration	The extent of integration of HBPM within a health system
	Sustainability	The extent to which HBPM is institutionalized within a health system or program
Health Service Outcomes [21]	Effectiveness	Providing HBPM based on scientific knowledge to all who could benefit and refraining from providing HBPM to those not likely to benefit (avoiding underuse and misuse, respectively)
	Efficiency	Avoiding waste of equipment, supplies, ideas, and energy
	Equity	Providing care that does not vary in quality because of personal characteristics such as gender, ethnicity, geographic location, and socioeconomic status
	Patient-centered	Providing care that is respectful of and responsive to individual patient preferences, needs, and values and ensuring that patient values guide all clinical decisions. Care that: (1) is respectful to patients’ values, preferences, and expressed needs; (2) is coordinated and integrated; (3) provides information, communication, and education; (4) ensures physical comfort; (5) provides emotional support—relieving fear and anxiety; and (6) involves family and friends
	Safety	Avoiding harm to patients from the care that is intended to help them
	Timeliness	Reducing waits and sometimes harmful delays for both those who receive and those who give care
Client Outcomes	Clinical status ^a^	Clinical status or symptomatology as result of or occurring after initiation of HBPM approach
	Function	Functioning in daily activities; social role functioning; and health promotion or prevention of adverse health outcomes
	Satisfaction	The extent to which a patient’s expectations about HBPM were met

^a^ The most relevant indicator of clinical status would be diagnosis of HDPs; however, given this was often criteria for HBPM eligibility, we did not consider it as an outcome measure.

**Table 3:** Characteristics of HBPM approaches identified in rapid review.

First Author and Year	Location	Patient Eligibility for HBPM	HBPM Approach: How BP Was Communicated to Health Providers
***Antepartum HBPM approaches***			
Lanssens 2018a [22] Lanssens 2018b [23] Lanssens 2019 [24]	Genk, Belgium	Pregnant and diagnosed with HDP	Uploaded via monitoring devices to digital platform
Pealing 2019 [25] Bowen 2021 [26] Pealing 2022 [27]	England	Pregnant and diagnosed with chronic or gestational hypertension, without pre-eclampsia	Uploaded via automated BP monitor
Tucker 2022 [28] Chappell 2022 [29] Campbell 2024 [30] Chisholm 2024a [31] Chisholm 2024b [32]	England	BUMP 1: Pregnant and at high risk for pre-eclampsia ^a^ BUMP 2: Pregnant and diagnosed with chronic hypertension or gestational hypertension	Uploaded via automated BP monitor Text to digital platform
Perry 2018 [33]	London, England	Pregnant and diagnosed with chronic hypertension, gestational hypertension or high risk of developing pre-eclampsia	Uploaded to digital platform
Xydopoulos 2019 [34]	London, England	Pregnant with history of pre-pregnancy hypertension, at risk of developing hypertension in pregnancy, systolic blood pressure ≥140 mmHg, diastolic blood pressure ≥90 mmHg, or proteinuria ≤1+	Uploaded to digital platform
Kalafat 2019 [35]	London, England	Pregnant and diagnosed with gestational hypertension	Uploaded to digital platform
Sheehan 2019 [36]	London, England	Not specified	Uploaded to digital platform
Lavallee 2024 [37]	Oxford, England	Pregnant and (1). diagnosed with hypertension or (2). at high risk of developing hypertension	Uploaded to digital platform
Postel-Vinay 2022 [38]	France	Pregnant	Uploaded to digital platform
Van den Heuvel 2019 [39]	Utrecht, Netherlands	Pregnant and low risk ^b^	Uploaded via automated BP monitor and sent to digital platform
Van den Heuvel 2020 [40] Jongsma 2020 [41] Van den Heuvel 2021 [42]	Utrecht, Netherlands	Pregnant and one or more risk factors for pre-eclampsia: chronic hypertension, pre-eclampsia in a prior pregnancy, maternal cardiac disease, or maternal kidney disease	Uploaded via automated BP monitor and sent to digital platform
Paterson 2023 [43]	Scotland	Pregnant and (1). high risk of hypertensive complication ^c^, (2). increased risk of developing pre-eclampsia ^d^, or (3). diagnosed with Type 1, or (4). carrying multiples	Uploaded to digital platform, 15 min telehealth appointments, Text or email to health providers
Jones 2023 [44] Jones 2024 [45]	Arkansas, United States	Pregnant and diagnosed with chronic hypertension	Uploaded via automated BP monitor to health provider portal
Howard 2024 [46]	Louisiana, United States	Pregnant	Uploaded via automated BP monitor to EHR
Runkle 2021 [47]	North Carolina, United States	Pregnant	Uploaded via automated BP monitor to health provider portal
Charifson 2024 [48]	Texas, United States	Pregnant	Uploaded via automated BP monitor or manually to digital platform
***Postpartum HBPM Approaches***			
Cairns 2018 [49] Cairns 2020 [50] Kitt 2021 [51]	England	Diagnosed with gestational hypertension or pre-eclampsia requiring anti-hypertensive treatment	Uploaded to digital platform or Text to digital platform
Runesha 2024 [52]	Illinois, United States	Diagnosed with HDP	Call to nursing call center
Mujic 2024 [53]	Massachusetts, United States	Diagnosed with chronic hypertension, gestational hypertension, pre-eclampsia, or de novo postpartum hypertension identified during the delivery hospitalization	Uploaded via automated BP monitor to EMR
Moustafa 2024 [54]	Mississippi, United States	Diagnosed with HDP	Text to study team (physicians)
Hayden-Robinson 2023 [55]	New York, United States	Diagnosed with HDP	Uploaded to patient portal Call to health provider
Hirshberg 2018 [56] Hirshberg 2019 [57] Triebwasser 2020 [58] Janssen 2021 [59]	Pennsylvania, United States	Diagnosed with pregnancy-related hypertension	Uploaded via automated BP monitor to digital platform and EMR
Burgess 2021 [60]	Pennsylvania, United States	Diagnosed with pre-eclampsia	Uploaded to digital platform
Burgess 2024 [61]	Pennsylvania, United States	Diagnosed with HDP	Uploaded to digital platform
Hauspurg 2019 [62] Lemon 2024 [63]	Pennsylvania, United States	Diagnosed with HDP	Uploaded via automated BP monitor to EHR
Reddy 2024 [64]	Pennsylvania, United States	Diagnosed with “persistent hypertension” at 6 weeks postpartum	Uploaded via automated BP monitor to EHR
Arkerson 2023 [65]	South Carolina, United States	Diagnosed with gestational hypertension, pre-eclampsia, or chronic hypertension with superimposed pre-eclampsia	Uploaded to digital platform
Hoppe 2019 [66] Hoppe 2020 [67] Thomas 2021 [68] Niu 2022 [69]	Wisconsin, United States	Diagnosed with HDP	Uploaded via automated BP monitor to digital platform
***Antepartum & Postpartum HBPM Approaches***			
Tran 2023 [70]	Vancouver, Canada	Risk factors for pre-eclampsia, diagnosed with HDP, or diagnosed with postpartum pre-eclampsia	Email to health provider
Wilson 2022 [71]	England	No standardized approach	No standardized approach
Bisson 2023 [72]	United States	No standardized approach	No standardized approach
Zhang 2024 [73]	Mississippi, United States	Pregnant	Uploaded via automated BP monitor to EHR

Abbreviations: BP: Blood Pressure; BUMP: Blood Pressure Monitoring in High-Risk Pregnancy to Improve the Detection and Monitoring of Hypertension; EHR: Electronic Health Records; HDPs: Hypertensive Disorders of Pregnancy; N/A: Not Applicable; ^a^ High risk for pre-eclampsia is defined as one or more of the following risk factors for pregnancy hypertension: age ≥40 years, nulliparity, pregnancy interval >10 years, family history of pre-eclampsia, previous history of pre-eclampsia or gestational hypertension, body mass index ≥30 kg/m^2^, chronic kidney disease, twin pregnancy, pre-pregnancy diabetes, or autoimmune disease. ^b^ Low risk defined as not having any of the following criteria: chronic hypertension, hypertensive disorder in a prior pregnancy, cardiac or renal pathology, obesity (BMI > 35), or arm circumference > 42 cm. ^c^ High risk defined as diagnosed with chronic hypertension, gestational hypertension, pre-eclampsia, cystic fibrosis, solid organ transplant, or cardiac conditions. ^d^ Increased risk defined as hypertensive disease during a previous pregnancy, diagnosed with chronic kidney disease, or diagnosed with autoimmune disease.

**Table 4:** Study design of research assessing HBPM approaches.

Author, Year	Study Design	Comparator	Primary Outcome
***Antepartum HBPM Approaches***			
Pealing 2019 [25]	RCT	Yes, usual care	Recruitment, retention, adherence and persistence with the intervention
Tucker 2022 [28]	RCT	Yes, usual care	Time to first recording of hypertension
Chappell 2022 [29]	RCT	Yes, usual care	Mean systolic BP measured by healthcare professionals between randomization and birth
Perry 2018 [33]	Case–control	Yes, usual care	Not specified
Van den Heuvel 2020 [40]	Case–control	Yes, usual care	Number of antenatal visits, ultrasounds, admissions and diagnostics
Kalafat 2019 [35]	Case–control	Yes, usual care	Adverse fetal, neonatal and maternal outcomes
Runkle 2021 [47]	Cohort	No	Retention and persistence of weekly BP monitoring during late-stage pregnancy
Howard 2024 [46]	Cohort	No	Average number of BP measurements during the prenatal and postpartum periods
Lanssens 2018a [22]	Cross-sectional	Yes, usual care	Number of prenatal consultations
Van den Heuvel 2019 [39]	Cross-sectional	No	Patient interaction and compliance
Bowen 2021 [26]	Cross-sectional	No	Proportion of days on which HBPM readings were taken
Postel-Vinay 2022 [38]	Cross-sectional	No	Not specified
Charifson 2024 [48]	Cross-sectional	No	Number of daily BP measures taken
Lavallee 2024 [37]	Cross-sectional	No	Not specified
Jongsma 2020 [41]	Mixed methods	No	Not specified
Jones 2023 [44]	Mixed methods	No	Not specified
Jones 2024 [45]	Mixed methods	Yes, pre/post	Not specified
Sheehan 2019 [36]	Qualitative	No	Not specified
Pealing 2022 [27]	Qualitative	No	Not specified
Paterson 2023 [43]	Qualitative	No	Not specified
Chisholm 2024a [31]	Qualitative	No	Not specified
Chisholm 2024b [32]	Qualitative	No	Not specified
Lanssens 2018b [23]	Cost analysis	No	Not specified
Lanssens 2019 [24]	Cost analysis	No	Not specified
Van den Heuvel 2021 [42]	Cost analysis	No	Cost per pregnancy of healthcare consumption
Campbell 2024 [30]	Cost analysis	No	Not specified
Xydopoulos 2019 [34]	Cost-minimization analysis	Yes, usual care	Number of outpatient visits, inpatient bed stays and investigations performed
***Postpartum*** ***HBPM Approaches***			
Cairns 2018 [49]	RCT	Yes, usual care	Recruitment, retention, and compliance with follow-up rates
Hirshberg 2018 [56]	RCT	Yes, usual care	Ascertainment of 1 BP in the first 10 days postpartum
Hirshberg 2019 [57]	Sub analysis of RCT	Yes, usual care	Ascertainment of 1 BP via office visit attendance or text
Kitt 2021 [51]	RCT	Yes, usual care	24 h average diastolic BP after 1 year postpartum
Arkerson 2023 [65]	RCT	Yes, usual care	Ascertainment of BP within 10 days postpartum
Hoppe 2020 [67]	Non-RCT	Yes, usual care	Number of hypertension-related hospital readmissions over 6-week postpartum period
Hoppe 2019 [66]	Cohort	No	Recruitment and retention in telehealth for postpartum hypertension
Triebwasser 2020 [58]	Cohort	Yes, trial vs. implementation groups	Ascertainment of 1 BP via text during the 10 days of monitoring
Janssen 2021 [59]	Cohort	No	Ascertainment of 1 BP via text in the 10 days of discharge
Lemon 2024 [63]	Cohort	Yes, usual care	Postpartum care utilization
Moustafa 2024 [54]	Cohort	No	Not specified
Hauspurg 2019 [62]	Cross-sectional	No	Engagement and retention through 42 days postpartum
Burgess 2021 [60]	Cross-sectional	No	Not specified
Thomas 2021 [68]	Cross-sectional	No	Not specified
Hayden-Robinson 2023 [55]	Cross-sectional	No	Not specified
Burgess 2024 [61]	Cross-sectional	No	Not specified
Runesha 2024 [52]	Cross-sectional	No	Not specified
Mujic 2024 [53]	Cross-sectional	No	Not specified
Reddy 2024 [64]	Cross-sectional	Yes, 6-week vs. extended monitoring groups	Not specified
Cairns 2020 [50]	Mixed methods	Yes, usual care	Not specified
Niu 2022 [69]	Cost-effectiveness analysis	Yes, usual care	Costs and QALYs
***Antepartum & Postpartum HBPM Approaches***			
Zhang 2024 [73]	Cohort	No	Adherence to prenatal protocol
Tran 2023 [70]	Cross-sectional	No	Provider use and interpretation of home BP to manage clinical care
Bisson 2023 [72]	Cross-sectional	No	Provider’s perception of HBPM utilization by patients
Wilson 2022 [71]	Mixed methods	No	Not specified

Abbreviations: BP: blood pressure; QALY: quality-adjusted life years; RCT: randomized control trial.

## Data Availability

No new data were created or analyzed in this study.

## Supplementary Materials

The following supporting information can be downloaded at: https://www.mdpi.com/article/10.3390/healthcare14081102/s1, Table S1: Search strategy; Table S2: Mapping of outcome measures for antepartum HBPM approaches; Table S3: Mapping of outcome measures for postpartum HBPM approaches; Table S4: Mapping of outcome measures for approaches that implement antepartum and postpartum HBPM.

## Abbreviations

The following abbreviations are used in this manuscript:

ACC	American College of Cardiology
AHA	American Heart Association
AJOG	American Journal of Obstetrics and Gynecology
BP	Blood Pressure
BUMP	Blood Pressure Monitoring in High-Risk Pregnancy to Improve the Detection and Monitoring of Hypertension
CIP	Community Implementation Program
ED	Emergency Department
EHR	Electronic Health Records
HBPM	Home Blood Pressure Monitoring
HDP	Hypertensive Disorder of Pregnancy
HRSA	Health Resources and Services Administration
MDMOM	Maryland Maternal Health Innovation Program
N/A	Not Applicable
QUALY	Quality-adjusted Life Years
RCT	Randomized Control Trial
