# Supplementary material for: Evaluation of Home Blood Pressure Monitoring for Patients with Hypertensive Disorders of Pregnancy: A Rapid Review
Source: Healthcare (Basel). 2026 Apr 20;14(8):1102. doi: 10.3390/healthcare14081102 (PMC13115600; doi:10.3390/healthcare14081102)
Supplement: Supplementary file 1 [file healthcare-14-01102-s001.zip › healthcare-4136245-supplementary.pdf]

Table S1: Search Strategy

Concept	Database	
	Scopus	MEDLINE
A. Home Blood Pressure Monitoring	“home blood pressure monitoring" OR "remote blood pressure monitoring" OR "self blood pressure monitoring" OR (“blood pressure monitoring” AND (home OR self OR remote OR telehealth))	“home blood pressure monitoring" OR "remote blood pressure monitoring" OR "self blood pressure monitoring"
B. Hypertensive disorders of pregnancy	"hypertensive disorders of pregnancy" OR "maternal health" OR "antenatal" OR "postpartum" OR “pregnancy”	"hypertensive disorders of pregnancy" OR "maternal health" OR "antenatal" OR "postpartum" OR “pregnancy”
A+B	385	152
Limited to years 2018-2024	251	115

Table S2: Mapping of outcome measures for antepartum HBPM approaches

Author, Year	Report of implementation outcomes								Report of health service outcomes						Report of client outcomes			Report of impact outcomes
	Acceptability	Adoption	Appropriatenes	Cost	Feasibility	Fidelity	Penetration	Sustainability	Effectiveness	Efficiency	Equity	Patient-centered	Safety	Timeliness	Clinical status	Function	Satisfaction	
**Experimental Designs**																		
Pealing 2019 (25)						■	■		■			■	■		■		■	■
Tucker 2022 (28)						■			■			■	■	■	■	■		■
Chappell 2022 (29)						■			■			■	■	■	■	■		■
**Observational Designs**																		
Perry 2018 (33)									■	■			■		■			■
Van den Heuvel 2020 (40)					■	■			■	■			■	■	■		■	■
Kalafat 2019 (35)										■			■		■			■
Runkle 2021 (47)					■	■			■				■					
Howard 2024 (46)						■					■							■
Lanssens 2018a (22)										■			■		■			■
Van den Heuvel 2019 (39)					■	■						■					■	
Bowen 2021 (26)						■			■									
Postel-Vinay 2022 (38)			■		■	■						■						
Charifson 2024 (48)						■												
Lavallee 2024 (37)		■			■	■	■		■									
**Mixed Methods**																		
Jongsma 2020 (41)			■		■					■		■		■			■	
Jones 2023 (44)			■		■							■					■	
Jones 2024 (45)			■		■	■						■	■	■	■		■	
**Qualitative**																		
Sheehan 2019 (36)									■			■					■	
Pealing 2022 (27)	■				■	■	■		■	■		■					■	

Author, Year	Report of implementation outcomes								Report of health service outcomes						Report of client outcome s			Report of impact outcomes
	Acceptability	Adoption	Appropriatenes	Cost	Feasibility	Fidelity	Penetration	Sustainability	Effectiveness	Efficiency	Equity	Patient-centered	Safety	Timeliness	Clinical status	Function	Satisfaction	
Paterson 2023(43)																		
Chisholm 2024a (31)																		
Chisholm 2024b (32)																		
**Economic Analyses**																		
Lanssens 2018b (23)																		
Lanssens 2019 (24)																		
Van den Heuvel 2021 (42)																		
Campbell 2024 (30)																		
Xydopoulos 2019 (34)																		
**TOTALS**	3	1	7	5	11	15	5	2	13	10	1	13	10	7	9	2	10	9

Table S3: Mapping of outcome measures for postpartum HBPM approaches

Author, Year	Report of implementation outcomes								Report of health service outcomes						Report of client outcomes			Report of impact outcomes
	Acceptability	Adoption	Appropriatenes	Cost	Feasibility	Fidelity	Penetration	Sustainability	Effectiveness	Efficiency	Equity	Patient-centered	Safety	Timeliness	Clinical status	Function	Satisfaction	
**Experimental designs**																		
Cairns 2018 (49)						■			■									
Hirshberg 2018 (56)			■			■			■				■				■	
Hirshberg 2019 (57)						■			■		■		■					
Kitt 2021 (51)																		■
Arkerson 2023 (65)			■		■	■			■	■	■		■				■	
Hoppe 2020 (67)						■			■				■	■				
**Observational designs**																		
Hoppe 2019 (66)					■	■			■				■	■			■	
Triebwasser 2020 (58)						■			■									
Janssen 2021 (59)			■		■	■			■			■	■				■	
Lemon 2024 (63)						■			■		■		■					
Moustafa 2024 (54)						■			■		■		■					
Hauspurg 2019 (62)					■	■			■			■	■				■	
Burgess 2021 (60)					■	■							■				■	
Thomas 2021 (68)					■							■	■				■	
Hayden-Robinson 2023 (55)					■	■			■				■					
Burgess 2024 (61)					■	■			■			■	■				■	
Runesha 2024 (52)			■		■	■			■			■	■				■	
Mujic 2024 (53)						■			■		■							
Reddy 2024 (64)									■			■						
**Mixed Methods**																		

Author, Year	Report of implementation outcomes								Report of health service outcomes						Report of client outcomes			Report of impact outcomes
	Acceptability	Adoption	Appropriatenes	Cost	Feasibility	Fidelity	Penetration	Sustainability	Effectiveness	Efficiency	Equity	Patient-centered	Safety	Timeliness	Clinical status	Function	Satisfaction	
Cairns 2020 (50)	■		■		■	■			■			■					■	
**Economic Analyses**																		
Niu 2022 (69)				■														
**TOTAL**	1	0	5	1	10	17	0	0	17	1	5	7	14	2	0	0	10	1

Table S4: Mapping of outcome measures for approaches that implement antepartum & postpartum HBPM

Author, Year	Report of implementation outcomes								Report of health service outcomes						Report of client outcomes			Report of impact outcomes
	Acceptability	Adoption	Appropriatenes	Cost	Feasibility	Fidelity	Penetration	Sustainability	Effectiveness	Efficiency	Equity	Patient-centered	Safety	Timeliness	Clinical status	Function	Satisfaction	
**Observational Designs**																		
Zhang 2024 (73)						■			■				■		■		■	■
Tran 2023 (70)					■	■	■		■			■					■	
Bisson 2023 (72)	■		■			■	■				■	■					■	
**Mixed Methods**																		
Wilson 2022 (71)					■	■			■	■		■					■	
**TOTAL**	1	0	1	0	2	4	2	0	3	1	1	3	1	0	1	0	4	1
